# Mogamulizumab-associated lymphadenopathy masquerading as lymphoma progression

**DOI:** 10.1016/j.jdcr.2026.01.046

**Published:** 2026-02-04

**Authors:** Parastou Tizro, Liliana Crisan, Karl Gaal, Steven T. Rosen, Jasmine Zain, Christiane Querfeld

**Affiliations:** a Department of Pathology, City of Hope Comprehensive Cancer Center, Duarte, California; b Division of Dermatology, City of Hope Comprehensive Cancer Center, Duarte, California; c Beckman Research Institute, City of Hope Comprehensive Cancer Center, Duarte, California; d Department of Hematology and Hematopoietic Cell Transplantation, City of Hope Comprehensive Cancer Center, Duarte, California

**Keywords:** cutaneous T-cell lymphoma, mogamulizumab, mogamulizumab-associated rash (MAR), reactive lymphadenopathy, Sézary syndrome, skin of color

Mogamulizumab is a monoclonal antibody approved by the Food and Drug Administration for relapsed/refractory cutaneous T-cell lymphoma (CTCL), mycosis fungoides, and Sézary syndrome (SS) subtypes. Mogamulizumab binds to C-C chemokine receptor 4 (CCR4) expressed on CTCL and regulatory T cells, leading to their selective depletion via immune-mediated cytotoxicity. While this enhances antitumor efficacy, improving progression-free survival and overall response rates, it may lead to immune-related adverse events that mimic disease progression.^1^, ^2^, ^3^ Mogamulizumab-associated rash (MAR), one of the most common side effects of mogamulizumab treatment, is well-recognized for its resemblance to CTCL lesions. Several studies indicate that the incidence of MAR is higher in patients who demonstrate a treatment response.^2^, ^3^, ^4^ Trum et al^2^ further noted that both MAR and clinical response may be more frequently observed in patients with a higher blood disease burden, such as those with SS, compared to mycosis fungoides. The underlying mechanism contributing to the increased frequency of MAR in SS and among responders is not fully understood, but is thought to be related to an enhanced immune response resulting from depletion of Tregs, activation of macrophage and production of CXCL9 and CXCL11 chemokines, and recruitment of CD8^+^ T cells to the skin.^5^ However, mogamulizumab-induced lymphadenopathy was reported sporadically and is not well characterized.

We present a patient with SS who responded to treatment but developed disseminated lymphadenopathy within 14 months of mogamulizumab initiation, mimicking disease progression and posing a significant diagnostic and therapeutic challenge.

## Case presentation

**Fig. 1:**
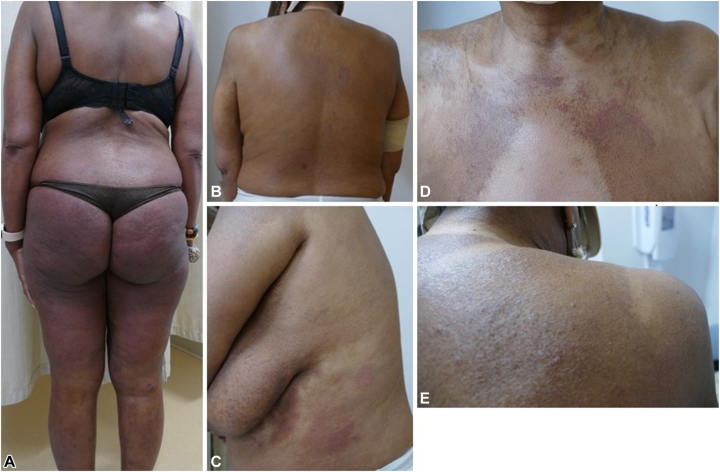
Clinical images of patient with Sézary syndrome with disseminated erythroderma at baseline **(A)** and clinical resolution at cycle 5 **(B)**. Development of MAR showing hypopigmented and mildly erythematous patches and admixed with hyperpigmenetd papules and plaques at cycle 10 **(C-E)**. *MAR*, Mogamulizumab-associated rash.

A 58-year-old Black female with SS, diagnosed 2 months prior, presented to our multidisciplinary cutaneous lymphoma clinic with generalized erythroderma (Fig 1, *A*) and disabling pruritus for clinical evaluation and management. Initial flow cytometry of peripheral blood showed 76% (27,640/μL) circulating Sézary cells (Table I). Skin biopsy with immunohistochemistry revealed an atypical CD4^+^ epidermotropic T-cell infiltrate with folliculotropism and syringotropism, and positivity for CCR4 in 30% of T cells consistent with CTCL. T-cell receptor (TCR) next-generation sequencing demonstrated identical TCR beta and gamma clones in skin and blood. Bone marrow biopsy was unremarkable. Computed tomography (CT) of the chest, abdomen, and pelvis showed multiple bilateral axillary and inguinal lymph nodes up to 14 mm in short axis. The diagnosis was stage IVA1 (T4NxM0B2b) SS. Initial antinuclear antibody screening for autoimmunity was negative.

**Table I:** Summary of results from peripheral blood flow cytometry and skin and lymph node biopsies, and imaging conducted at baseline, and at the onset of mogamulizumab-associated rash and reactive lymphadenopathy

Study	Baseline	Mogamulizumab-associated rash	Mogamulizumab-associated lymphadenopathy
Flow cytometry (peripheral blood)	• 27,640 cells/μL • CD4:CD8 ratio: 95 • CD4^+^/CD26^−^: 95%	• 1,168 cells/μL • CD4:CD8 ratio: 9.4 • CD4^+^/CD26^−^: 75%	• 2,464 cells/μL • CD4:CD8 ratio: 30 • CD4^+^/CD26^−^: 77%
Histology with immunohistochemistry	• Atypical epidermotropic lymphoid infiltrate with adnexotropism • CD20^−^, CD3^+^, CD4^+^, CD8^−^, CD5^+^, CD7^+/−^, PD1^+^, CD30∼5% • CD4:CD8 ratio >10:1	• Spongiotic dermatitis and lymphoid atypia consistent with MAR and residual CTCL • CD20^−^, CD3^+^, CD4^+^, CD8^−^, CD7^−^ • CD4:CD8 ratio∼10:1	• Follicular hyperplasia and dermopathic changes • CD20^+^, CD3^+^, CD4^+^, CD8^+^ (scattered, paracortical & interfollicular), preserved CD5^+^ and CD7^+^ expression, CD30^+^ (scattered), PD1^+^ (Tfh subset), CD68^+^, and scattered FOXP3^+^ cells • Normal CD4:CD8 ratio
Dominant TCR gene segments	• Identical TCR*β* (Vb6-5/Jb2-2 & Db1/Jb2-7) and TCRɣ (Vg10/JgP1) in blood and skin	• TCR*β* (Vb6-5/Jb2-2 & Db1/Jb2-7) in skin below the threshold for defining a clonal population consistent with minimal residual disease • Low clonal abundance of TCRɣ (Vg10/JgP1) in skin	• Low abundance of new TCR*β* (Vb12-4/Jb1-2 & Db1/Jb1-3) in LN, with absence of TCR*β* picks observed in blood/skin • Low abundance of TCRɣ (Vg10/JgP1) in LN (<5%)
Imaging	• CT chest, abdomen, and pelvis revealed bilateral axillary lymphadenopathy up to 14 mm in short axis. Multiple bilateral inguinal LN presented but not enlarged by CT size criteria	• Not performed	• PET/CT scans with extensive, peripheral and central, FDG-avid lymphadenopathy up to 41 mm in long axis and 27 mm in short axis, with SUV ranging from 4.7 to 11.6

*CT*, Computed tomography; *FDG*, fluorodeoxyglucose; *LN*, lymph node; *PET*, positron emission tomography; *TCR*, T-cell receptor; *Tfh*, follicular helper T cells; *SUV*, standardized uptake value.

**Fig. 2:**
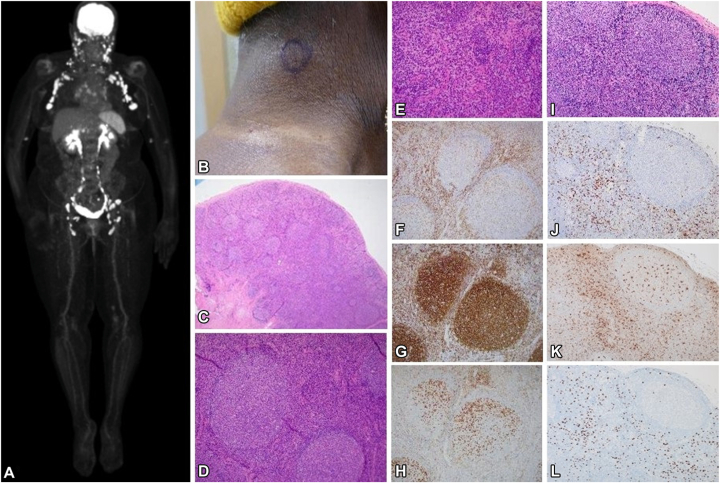
PET-CT showing widespread FDG-avid lymphadenopathy **(A).** initially raising concern for disease progression during mogamulizumab treatment. Clinical finding of cervical lymphadenopathy **(B)**. Right cervical node biopsy revealing follicular hyperplasia composed of numerous follicles with large, irregular germinal centers, and retained mantle zones **(C, D)**. Paracortical area with numerous histiocytes with focal pigmentation consistent with dermopathic changes **(E)** [hematoxylin-eosin stain, ×20, ×100, and ×200 magnification]. Immunophenotyping demonstrating a moderate number of CD3^+^ T cells **(F)**. CD20 immunostain highlighting B cells in germinal centers **(G),** with PD1 highlighting T-follicular helper cells **(H)** [×100 magnification]. Additional sections demonstrate paracortical and interfollicular expansion of small lymphocytes and histiocytes on H&E **(I),** with corresponding immunohistochemistry stains for CD8 **(J),** CD68 **(K),** and FOXP3 showing T-regulatory cells **(L)** [×200 magnification]. (*CT*, Computed tomography; *PET*, positron emission tomography.

The patient had used topical steroids and weekly low-dose methotrexate before presentation. Mogamulizumab was started weekly (4 doses), then biweekly of a 28-day cycle. After 2 cycles, a nearly complete remission in blood and partially improved erythroderma was noted. Over subsequent cycles, erythema (Fig 1, *B*) and pruritus subsided. At cycle 10, recurrent pruritus and new erythematous, scaly, and vitiligo-like, hypopigmented skin patches developed in the patient (Figs 1, *C-E*). Skin biopsy showed spongiotic dermatitis with focal T-cell atypia interpreted as MAR with residual CTCL. Mogamulizumab was paused for 2 weeks, and topical steroids were administered leading to significant improvement. At cycle 15, new palpable peripheral lymphadenopathy was noted without active skin lesions. Positron emission tomography (PET)/CT scans revealed numerous new and increased metabolically active fluorodeoxyglucose (FDG)-avid nodes up to 27 mm in short axis and standardized uptake value up to 11.6, located bilateral occipital, preauricular, intraparotid, cervical, axillary, mediastinal, hilar, upper abdominal, pelvic, and bilateral elbow-region (Fig 2, *A*). Both fine-needle and excisional biopsy of bilateral level III/V cervical nodes showed reactive follicular and paracortical hyperplasia consistent with dermopathic changes, negative for CTCL (Figs 2, *B-D*). Immunohistochemistry showed a mixed T-cell population with normal CD4:CD8 ratio and numerous macrophages within the paracortical area. Concurrently, TCR sequencing identified a new TCRβ clone with persistent but low clonal abundance for TCRγ. Mogamulizumab was held and low-dose methotrexate resumed. Lymphadenopathy resolved over 3 months and PET/CT scans showed focally measurable and minimally metabolically active disease, although Sézary cell counts slowly rebounded. Given the limited treatment options for CTCL and patient’s significant improvement in quality of life without definite disease progression, mogamulizumab was reintroduced (biweekly) with methotrexate, producing sustained skin remission and preventing recurrent lymphadenopathy for over 8 months, despite moderate blood involvement.

## Discussion

Mogamulizumab has demonstrated a favorable safety profile in patients with CTCL, with infusion reactions, rash, diarrhea, and fatigue being the most common adverse events. In the pivotal MAVORIC trial, MAR was a side effect reported in approximately 24% of MF/SS patients, while reactive lymphadenopathy was not specifically reported. Extracutaneous disease assessment was primarily conducted by CT imaging, which might not recognize reactive lymphadenopathy.^1^ To date, only 2 cases of mogamulizumab-induced lymphadenopathy have been reported.^6^ Whether this rarity reflects under-recognition or truly low prevalence remains unclear, as nodal enlargement is frequently attributed to disease progression without biopsy confirmation. Peripheral lymphadenopathy is frequent in SS; however, new-onset central lymphadenopathy without visceral involvement remains uncommon.^7^

In this case, the absence of atypical T cells, normal CD4:CD8 ratio, discordant TCR*β* clonotype between lymph node and blood/skin compartment, resolution of lymphadenopathy after mogamulizumab discontinuation, and lack of systemic symptoms support a drug-induced lymphadenopathy rather than CTCL progression. In addition, the histologic findings of preserved architecture with florid B-cell–rich germinal centers and follicular hyperplasia favor a reactive or drug related lymphadenitis rather that CTCL involvement, in which germinal centers are typically attenuated with paracortical expansion of atypical CD4^+^ T cells. Unlike the patients reported by Calderón-Lozano et al,^6^ who developed MAR and lymphadenopathy concurrently, our patient presented with lymphadenopathy 5 months after onset of M AR presenting as a partially vitiligo-like and/or scaly skin eruption, and lymph node histology exhibited focal paracortical hyperplasia in addition to follicular hyperplasia. Notably, mixed follicular and parafollicular hyperplasia was previously reported in cases of drug-induced lymphadenopathy.^8^ Taken together, comparison with the 2 published cases by Calderón-Lozano et al^6^ (Table II) suggests that mogamulizumab-induced lymphadenopathy can arise at different time points during therapy and display variable histologic and molecular features, yet consistently exhibits intense central and peripheric FDG uptake and a benign, reversible course once the drug is withheld.

**Table II:** Comparative timeline and histological and imaging pattern of mogamulizumab-associated lymphadenopathy in all reported cases to date

Features	Case 1 (Calderón-Lozano et al^6^)	Case 2 (Calderón-Lozano et al^6^)	Present case
CTCL subtype	Mycosis fungoides (T4N0M0B2)	Mycosis fungoides (T4N0M0B2)	Sézary syndrome (T4NxM0B2b)
Time to MAR onset	Cycle 18	Cycle 7	Cycle 10
Time to detection of lymphadenopathy	Concurrent with MAR	Concurrent with MAR	MAR preceding LAD by ∼5 months
PET/CT activity	FDG avid	FDG avid	FDG avid
LN histology	Nonnecrotizing granulomatous lymphadenitis	Reactive follicular hyperplasia	Follicular hyperplasia and dermopathic changes
TCR rearrangement	Negative	Negative	Low abundance of new TCR*β*, low abundance of TCRɣ

*CT*, Computed tomography; *CTCL*, cutaneous T-cell lymphoma; *FDG*, fluorodeoxyglucose; *LAD*, lymphadenopathy; *LN*, lymph node; *MAR*, mogamulizumab-associated rash; *PET*, positron emission tomography; *TCR*, T-cell receptor.

Given that CCR4 depletes T regs but also binds the dendritic cell- and macrophage-derived ligands CCL17 and CCL22,^9^ we hypothesize that the pathomechanism of mogamulizumab-associated lymphadenopathy may parallel, at least in part, that of MAR and involves both loss of immune regulation and rebound macrophages activation. The lymph node histology, with abundance of CD68^+^ macrophages, supports our hypothesis. CD8^+^T cells were present as scattered interfollicular (Fig 2), which suggests that CD8 predominance is less evident in mogamulizumab-associated lymphadenopathy than previously reported in MAR.

## Conclusion

Recognition of mogamulizumab-induced lymphadenopathy is essential to prevent misdiagnosis of CTCL progression and avoid unnecessary discontinuation in therapy. The treating clinical team should remain vigilant for this benign, reversible phenomenon, particularly in patients presenting with new central lymphadenopathy during treatment. The increased metabolic activity on PET/CT scans can mimic CTCL progression; hence, a timely histopathological evaluation and consideration of drug-induced mechanisms can facilitate accurate diagnosis, optimize patient management, and support continued use of effective therapies in CTCL. The molecular findings of TCR sequencing analysis further substantiates the clinical and histopathologic evidence for a benign, reversible mogamulizumab-induced lymphadenopathy rather than active CTCL progression. Low-dose weekly oral methotrexate may mitigate immune reactions and enable a successful rechallenge with mogamulizumab.

## Conflicts of interest

Dr Querfeld is in Advisory board/steering committee, Kyowa Kirin, Helsinn, Citius Pharmaceuticals, SLAM BioTherapeutics (research grants: Kyowa Kirin and Helsinn). Drs Tizro, Crisan, Gaal, Rosen, and Zain have no conflicts of interest to declare.
